# Depletion of Survivin suppresses docetaxel-induced apoptosis in HeLa cells by facilitating mitotic slippage

**DOI:** 10.1038/s41598-021-81563-3

**Published:** 2021-01-27

**Authors:** Teng-Long Han, Hang Sha, Jun Ji, Yun-Tian Li, Deng-Shan Wu, Hu Lin, Bin Hu, Zhi-Xin Jiang

**Affiliations:** The 305 Hospital of the People’s Liberation Army, Beijing, 100017 China

**Keywords:** Cancer therapeutic resistance, Apoptosis

## Abstract

The anticancer effects of taxanes are attributed to the induction of mitotic arrest through activation of the spindle assembly checkpoint. Cell death following extended mitotic arrest is mediated by the intrinsic apoptosis pathway. Accordingly, factors that influence the robustness of mitotic arrest or disrupt the apoptotic machinery confer drug resistance. Survivin is an inhibitor of apoptosis protein. Its overexpression is associated with chemoresistance, and its targeting leads to drug sensitization. However, Survivin also acts specifically in the spindle assembly checkpoint response to taxanes. Hence, the failure of Survivin-depleted cells to arrest in mitosis may lead to taxane resistance. Here we show that Survivin depletion protects HeLa cells against docetaxel-induced apoptosis by facilitating mitotic slippage. However, Survivin depletion does not promote clonogenic survival of tumor cells but increases the level of cellular senescence induced by docetaxel. Moreover, lentiviral overexpression of Survivin does not provide protection against docetaxel or cisplatin treatment, in contrast to the anti-apoptotic Bcl-xL or Bcl-2. Our findings suggest that targeting Survivin may influence the cell response to docetaxel by driving the cells through aberrant mitotic progression, rather than directly sensitizing cells to apoptosis.

## Introduction

Docetaxel and paclitaxel are taxanes that are widely used in the clinic for the treatment of ovarian, breast, lung cancers and other solid tumors^1^. They share a common mechanism of action by promoting and stabilizing microtubule assembly, which disrupts microtubule dynamics and chronically activates the spindle assembly checkpoint (SAC), thereby inducing an extended mitotic arrest that ultimately leads to cell death^2^. Premature mitotic exit, also known as mitotic slippage, usually due to a defective SAC is considered a process through which tumor cells evade killing by taxanes and develop drug resistance^3^. Although non-mitotic functions of taxanes have been reported and paclitaxel causes interphase cell death^4^, pro-apoptotic signals frequently accumulate during extended mitotic arrest^5^. Moreover, anti-apoptotic proteins often decrease or lose their pro-survival functions during mitotic arrest^6^, indicating a causal relationship between mitotic arrest and apoptosis. Accordingly, targeting mitotic exit^7^ or anti-apoptotic proteins^8^ has been shown to enhance the anticancer activity of taxanes.

Survivin is a 16.5 kD member of the inhibitor of apoptosis (IAP) family which is thought to function in both apoptosis inhibition and mitosis regulation^9^. Unlike other IAPs, Survivin has clear cell cycle-dependent expression at mitosis and is degraded by the ubiquitin–proteasome pathway in the G1 phase of the cell cycle^10^. Together with Aurora-B kinase, inner centromere protein (INCENP), and Borealin/Dasra, Survivin is a component of the chromosome passenger complex (CPC), which is essential for proper chromosome segregation and cytokinesis^11^. Depletion of Survivin invariably results in aberrant mitotic progression^12^, and its mitotic phosphorylation by p34cdc2-cyclin B1 has been associated with increased stability^13^.

The role of Survivin in apoptosis inhibition has been a subject of debate. Although Survivin is unable to directly bind to and inhibit caspase activity similar to other IAPs^14^, the overexpression of Survivin is associated with the inhibition of cell death induced by multiple anticancer agents including taxanes^15, 16^. Furthermore, Survivin is undetectable in normal adult tissues but is selectively expressed in transformed cells and in most human cancers, making it a promising therapeutic target for the treatment of cancer^10^. Targeting Survivin by using antisense nucleotides, dominant-negative mutants, ribozymes, or RNA interference (RNAi) results in spontaneous caspase-dependent cell death^17^ and tumor sensitization to chemotherapeutic agents, such as paclitaxel^18^, docetaxel^19^ and cisplatin^20^. However, as a key regulator of mitosis, Survivin is required for the maintenance of the SAC when it is activated by paclitaxel^21^. Depletion of Survivin results in failure of sustained mitotic arrest induced by paclitaxel, which may potentially increase cancer cell survival.

In this study, we sought to clarify the role of Survivin in the cellular response to docetaxel, accomplished through lentiviral vector-mediated Survivin overexpression and small interfering RNA (siRNA)-mediated Survivin depletion. The results show that Survivin-depleted cancer cells undergo aberrant mitotic progression which results in the induction of apoptosis. Depletion of Survivin facilitates mitotic slippage of HeLa cells in response to docetaxel treatment and reduces drug-induced apoptosis. Furthermore, overexpression of Survivin does not block apoptosis induced by docetaxel or cisplatin trreatment, in contrast to overexpression of Bcl-xL or Bcl-2, suggesting that the anti-apoptotic function of Survivin is negligible.

## Results

### Apoptosis following docetaxel treatment is closely linked with mitotic arrest

**Figure 1:**
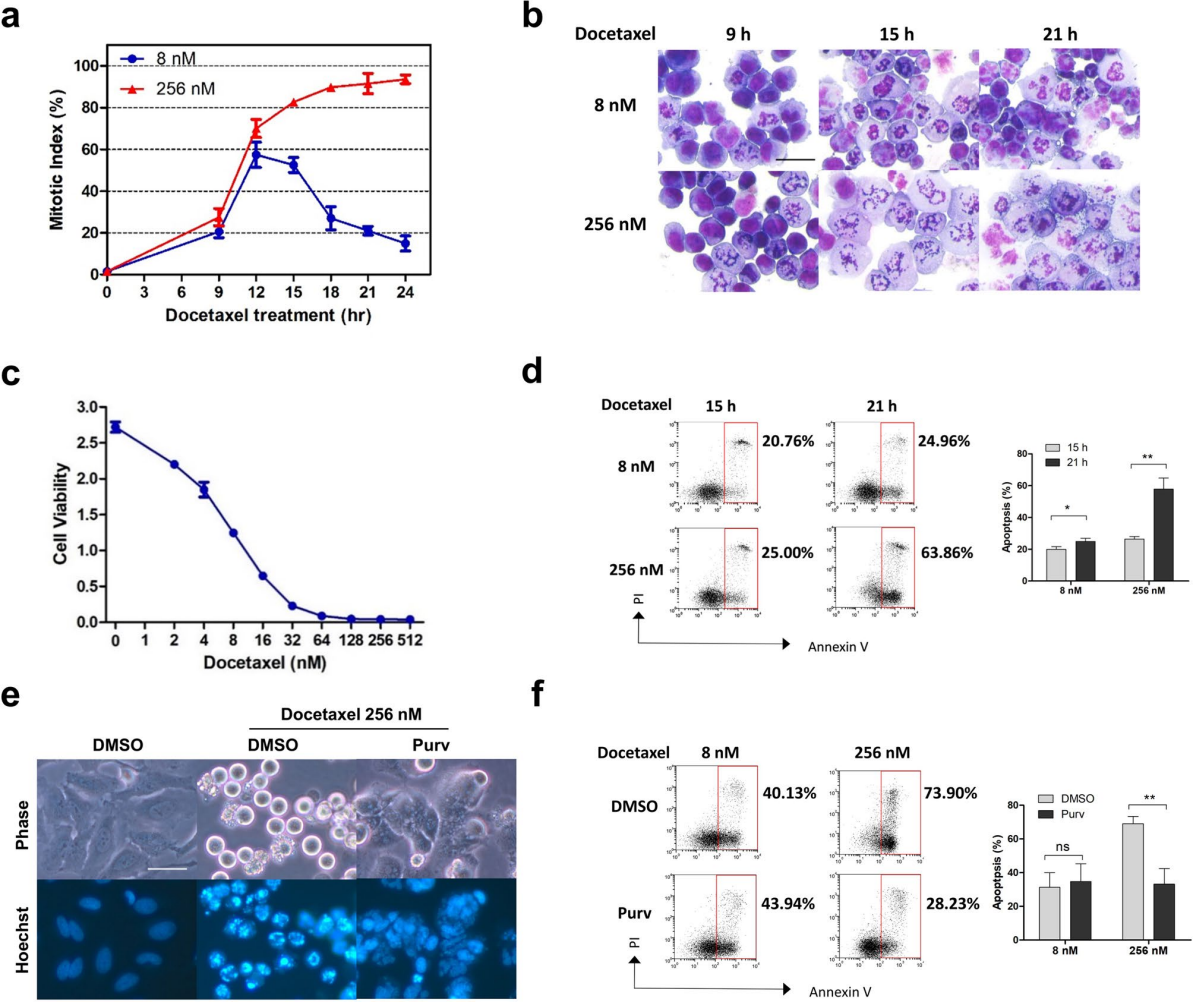
Association between docetaxel-induced mitotic arrest and apoptosis. (**a**,**b**) HeLa cells were synchronized at the G1/S boundary by double-thymidine block. Upon release from the block (0 h), the cells were treated with indicated concentrations of docetaxel, collected at indicated time intervals and subjected to Giemsa staining. Mitotic indexes (**a**) were determined by counting the percentage of cells with condensed chromatin. Values represent mean ± s.d. (n = 3 fields). Representative fields of docetaxel-treated HeLa cells at the indicated time points are shown in panel (**b**). (**c**) Cell viability assay. HeLa Cells were treated with indicated increasing concentrations of docetaxel. Cell viability was determined 3 days following treatment by using the cck-8 reagent. Values represent mean ± s.d. (n = 3 wells). (**d**) HeLa cells were synchronized by double-thymidine block, released into fresh medium containing indicated concentration of docetaxel, harvested at indicated time points and stained for Annexin V/PI. Annexin V-positive (apoptotic) cells were analyzed by FACS. The percentage of apoptotic cells is shown. Values represent mean ± s.d. from a duplicate experiment. (**e**,**f**) HeLa cells were synchronized by double-thymidine block, released into medium containing indicated concentration of docetaxel for 16 h and treated with either DMSO or Purv (10 μM) in the presence of docetaxel. 4 h after the addition of Purv, cells were stained with Hoechst 33342 and photographed using phase and Hoechst fluorescence (**e**). Alternatively, 8 h after the addition of Purv, cells were collected or analyzed for induction of apoptosis (**f**). The percentage of apoptotic cells is shown. Values represent mean ± s.d. from a duplicate experiment. *Signifies p < 0.05; **p < 0.01; ns, not significant. Unpaired and two-tailed t test was used. Scale bars in (**b**) and (**e**), 50 μm.

Taxane treatment leads to mitotic arrest and apoptosis of most types of cancer cells. To determine the robustness of mitotic arrest, we measured the mitotic index of SAC-proficient HeLa cells^22^ after challenge with different concentration of docetaxel. While 256 nM docetaxel efficiently arrested the cells in mitosis, cells treated with 8 nM were blocked only transiently. After 24 h of treatment, cells treated with 256 nM docetaxel exhibited a mitotic index greater than fourfold more than those treated with 8 nM (Fig. 1a). At 15 h, most cells in response to 8 or 256 nM docetaxel exhibited typical mitotic morphology with condensed chromatin. At 21 h, however, most cells in response to 8 nM slipped out of mitosis, as determined by chromosome de-condensation and multi-nucleation (Fig. 1b). These findings indicate that the duration of mitotic arrest following docetaxel treatment depends on drug concentration.

Consistent with previous findings, high concentrations of docetaxel led to more significant cell death compared with low concentrations (Fig. 1c). To determine whether the fraction of cell death induced by docetaxel correlated with the duration of mitotic arrest, we compared the level of apoptosis in cells undergoing extended versus transient mitotic arrest. Treatment with 256 nM Docetaxel, which led to sustained mitotic arrest, resulted in a marked increase in the level of apoptosis during 15–21 h treatment. In contrast, the level of cell death caused by 8 nM docetaxel, which block mitosis transiently, was only slightly elevated during the same time period (Fig. 1d).

To determine the causal relationship between extended mitotic arrest and apoptosis, HeLa cells were forced to exit docetaxel-induced mitosis arrest by the addition of purvalanol A (Purv), which inhibits the mitotic kinase p34cdc2, thus facilitating mitotic slippage in the presence of activated SAC^23^. In contrast to the spherical cells with condensed chromatin induced by 256 nM docetaxel alone, sequential addition of Purv yielded large cells with multiple micronuclei and de-condensed chromatin (Fig. 1e), suggesting that these cells had exited mitosis without proper cell division. Notably, Purv administration greatly reduced the level of apoptosis induced by 256 nM docetaxel, but did not prevent cell death caused by 8 nM (Fig. 1f), indicating that the protective effect resulted from the abrogation of mitotic arrest. Together, these findings support the correlation between docetaxel-induced mitotic arrest and apoptosis, and suggest that mitotic slippage could be a mechanism that promotes cancer cell survival against docetaxel treatment.

### Overexpression of Survivin does not cause resistance to docetaxel or cisplatin treatment

**Figure 2:**
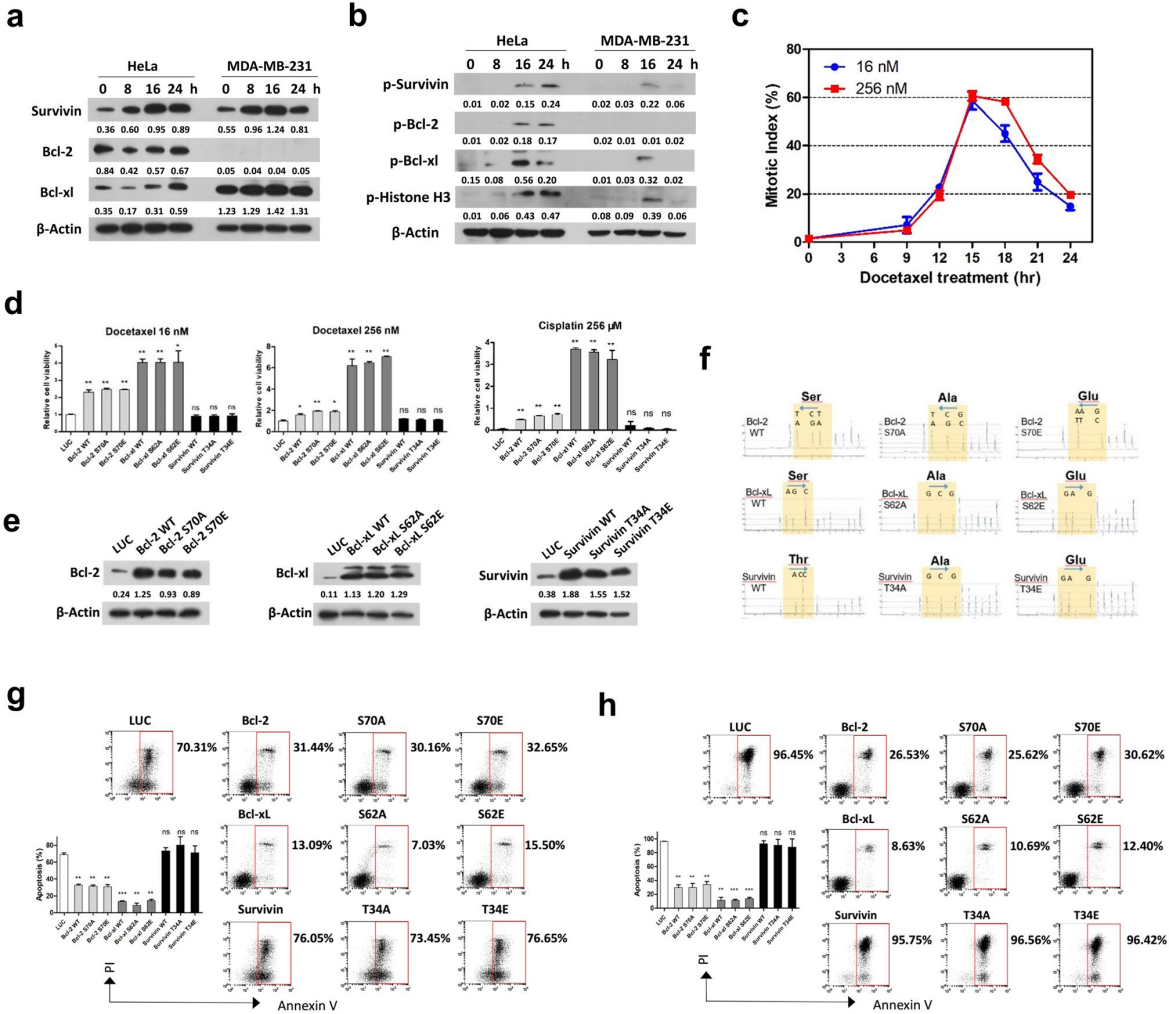
Lentiviral overexpression of Survivin does not confer chemoresistance. (**a**,**b**) HeLa and MDA-MB-231cells were synchronized by double-thymidine block, released into medium containing 256 nM docetaxel and harvested at the indicated time intervals. Protein expression (**a**) or phosphorylation (**b**) of Survivin, Bcl-2, and Bcl-xL following docetaxel treatment was determined by western blotting. Phosphorylation of histone H3 was used as a mitotic marker. The band intensities were measured using ImageJ software and normalized to the β-actin. Original blots can be found in Supplementary Fig. S1. (**c**) MDA-MB-231 cells were synchronized by double-thymidine block, released into fresh medium containing indicated concentration of docetaxel and harvested at indicated time points. Mitotic indexes were determined by counting the percentage of cells with condensed chromatin. Values represent mean ± s.d. (n = 3 fields). (**d**) HeLa cells were infected with lentivirus expressing the indicated proteins for 48 h, followed by docetaxel or cisplatin treatment for another 48 h before cell viability was determined. Values represent mean ± s.d. (n = 2 wells). (**e–h**) HeLa cells stably expressing wild-type or mutant forms of Bcl-2, Bcl-xL, or Survivin were generated by lentivirus infection followed by selection with puromycin. Expression of indicated protein in stable cell lines was validated by western blotting (**e**). The band intensities were measured using ImageJ software and normalized to the β-actin. Original blots can be found in Supplementary Fig. S1. Expression of mutant protein was validated by cDNA pyrosequencing (**f**). Stable cells expressing the indicated protein were treated with docetaxel (256 nM) (**g**) or cisplatin (256 μM) (**h**), then collected and analyzed for induction of apoptosis (**g**,**h**). The percentage of apoptotic cells are shown. Values represent mean ± s.d. from a duplicate experiment. *Signifies p < 0.05; **p < 0.01; ***p < 0.001; ns, not significant. Unpaired and two-tailed t test was used.

Overexpression of anti-apoptotic proteins is frequently involved in chemoresistance. The anti-apoptotic function of Bcl-2 or Bcl-xL is reported to be abrogated through mitotic phosphorylation^24^. However, the anti-apoptotic function of Survivin depends on mitotic phosphorylation to maintain its stability^13^. To determine the effect of mitotic phosphorylation on anti-apoptotic function of Bcl-2, Bcl-xL and Survivin, we compared endogenous expression (Fig. 2a) and phosphorylation (Fig. 2b) of these proteins in HeLa and MDA-MB-231 cells following treatment with 256 nM docetaxel. Unlike HeLa cells, MDA-MB-231 cells were incapable of sustaining the mitotic arrest caused by either 16 or 256 nM docetaxel (Fig. 2c), suggesting a weakened SAC. In line with the mitosis-specific expression^15^, the protein level of Survivin increased following docetaxel treatment, peaking at 16 h and maintained at an approximate level at 24 h in HeLa cells, consistent with the duration of mitotic arrest (Fig. 2a). In contrast, Survivin protein in MDA-MB-231 cells markedly decreased at 24 h, when most cells had undergone mitotic exit. The protein level of Bcl-2 was negligible in MDA-MB-231 cells which express a markedly higher level of Bcl-xL compared with HeLa cells. Phosphorylation of these proteins was exclusively observed in cells that were arrested in mitosis (Fig. 2b).

To express these proteins effectively, we took advantage of a lentiviral expression system (detailed in the “Methods” section), which circumvents the cytotoxicity mediated by plasmid transfection and shows premium gene transfer efficiency. HeLa cells were transduced with lentiviral particles expressing wild-type (WT) (Bcl-2, Bcl-xL and Survivin), phospho-defective (Bcl-2-S70A^25^, Bcl-xL-S62A^24^ and Survivin-T34A^26^) or phosphor-mimetic (Bcl-2-S70E^25^, Bcl-xL-S62E^27^ and Survivin-T34E^28^) mutant proteins. Lentivirus expressing luciferase gene (LUC) was used as a control. We found that cells infected with lentiviral particles expressing Bcl-2 or Bcl-xL were significantly more resistant to docetaxel or cisplatin treatment compared with control cells (Fig. 2d). Bcl-xL was more protective than Bcl-2, in accordance with its more potent anti-apoptotic function^29^. Notably, mutation at the phosphorylation site did not significantly affect the anti-apoptotic function of Bcl-2 or Bcl-xL. Contrary to expectations, transduction with lentivirus expressing Survivin, whether WT, phospho-defective or phospho-mimetic mutant, did not show any protective effect against docetaxel or cisplatin treatment.

To further test the role of these proteins in cellular response to therapy, stable cell lines expressing WT or mutant forms of Bcl-2, Bcl-xL, or Survivin were generated by selecting lentivirus-infected cells with puromycin. Overexpression of the indicated proteins was validated by western blotting (Fig. 2e), and point mutation at the phosphorylation site was validated by pyrosequencing the cDNA of stable cell lines (Fig. 2f). A substantial decrease in docetaxel- (Fig. 2g) or cisplatin-mediated (Fig. 2h) cell death was observed in stable cells overexpressing Bcl-xL or Bcl-2. Expression of phospho-defective mutant of Bcl-xL (S62A) showed a slightly better protective effect against docetaxel treatment over WT protein (Fig. 2g). However, the anti-apoptotic activities were largely retained in phospho-mimetic mutants, indicating that the impact of mitotic phosphorylation on anti-apoptotic function of Bcl-xL or Bcl-2 was minimal. These observations are in line with a previous study^30^. Notably, overexpression of WT Survivin or its phospho-mimetic or deficient mutants was not protective against either docetaxel or cisplatin treatment, in contrast to anti-apoptotic Bcl-xL or Bcl-2.

### Survivin depletion causes apoptosis through abnormal mitosis

**Figure 3:**
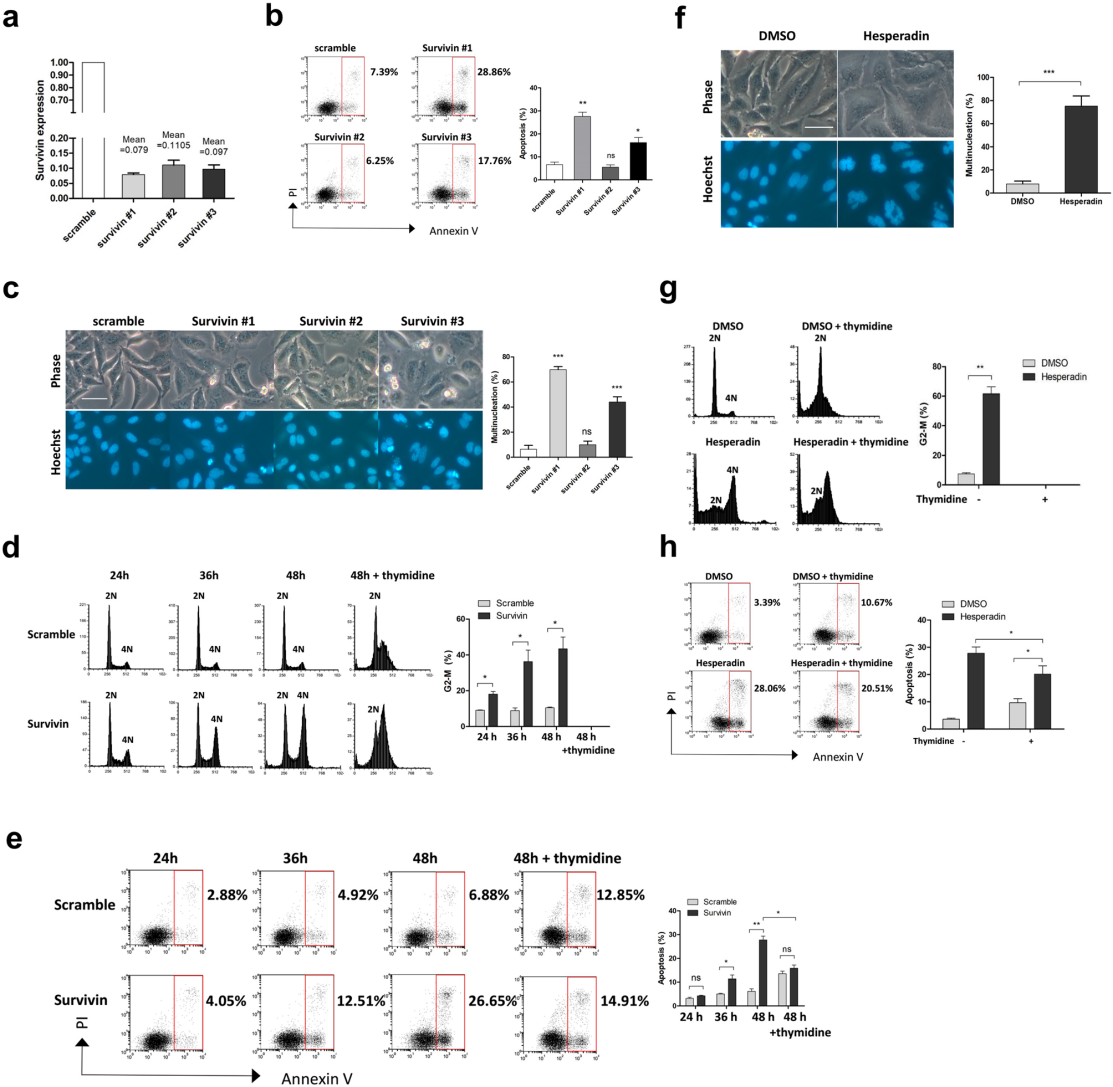
Apoptosis following Survivin depletion is associated with abnormal mitosis. (**a-c**) HeLa cells were transfected with a scramble control siRNA or with three previously validated siRNAs targeting Survivin for 48 h, respectively. The knockdown efficiency of RNAi was determined by real-time PCR analysis (**a**). Induction of apoptosis was determined by Annexin V/PI staining and FACS analysis (**b**). Alternatively, cells were stained with Hoechst 33342 and photographed using phase and Hoechst fluorescence (**c**). (**d**,**e**) HeLa cells were incubated with control or Survivin siRNA for the indicated time and then collected and either assessed for cell cycle distribution by PI staining followed by FACS analysis (**d**) or processed for the assessment of apoptosis (**e**). Alternatively, cells were incubated with thymidine (2 mM) along with the indicated siRNA for 48 h and then collected and assessed for either cell cycle distribution (**d**) or induction of apoptosis (**e**). (**f–h**) HeLa cells were treated with DMSO or Hesperadin (50 nM) for 36 h and then either stained with Hoechst 33342 and photographed using phase and Hoechst fluorescence (**f**), or processed for the detection of cycle distribution (**g**) or induction of apoptosis (**h**). Alternatively, cells were incubated with thymidine (2 mM) for 12 h, followed by treatment with either DMSO or Hesperadin (50 nM) for 36 h in the presence of thymidine. Cells were then collected and assessed for either cell cycle distribution (**g**) or induction of apoptosis (**h**). The percentage of apoptotic or multinucleated cells or cells in G2-M phase is shown. Values represent mean ± s.d. *Signifies p < 0.05; **p < 0.01; ***p < 0.001; ns, not significant. Unpaired and two-tailed t test was used. Scale bars in c and f, 50 μm.

Next, we determined whether Depletion of Survivin resulted in spontaneous apoptosis as described in previous literature^17^. Considering the difference in downregulation efficiency between siRNAs, three distinct, previously validated siRNAs^21, 31, 32^ were used for Survivin targeting. Transfection of each siRNA resulted in the successful silencing of Survivin, with the downregulation efficiency ranging from ~ 89% to ~ 92%, as shown by quantitative PCR analysis (Fig. 3a). Transfection of siRNA #1, which showed the highest knockdown efficiency (~ 92% downregulation), resulted in the largest amount of cell death (~ 29% apoptosis) (Fig. 3b). Induction of apoptosis was also observed following transfection of siRNA #3 (~ 90% downregulation, ~ 18% apoptosis). However, the level of apoptosis induced by siRNA #2 (~ 89% downregulation, ~ 6% apoptosis) was comparable to that of scramble control siRNA (~ 7% apoptosis), suggesting that a critical minimum threshold level of Survivin is required for cell survival. Depletion of Survivin has also been linked to abnormal cytokinesis^12^. Accordingly, transfection of siRNA #1 or #3 resulted in a significant increase of multinucleated cells (Fig. 3c). However, most of the cells transfected with siRNA #2 were mononuclear, similar to the control cells. The siRNA #1 with the highest downregulation efficiency was used in subsequent experiments.

To determine whether the apoptotic phenotype following Survivin knockdown was the result of aberrant mitosis, HeLa cells transfected with Survivin or control siRNA were harvested at 12 h intervals and assessed for cell cycle distribution (Fig. 3d) and induction of apoptosis (Fig. 3e). A 24 h incubation with Survivin siRNA led to a modest increase in the population with 4 N DNA content without significant induction of apoptosis. As 4 N population increased over time, the fraction of apoptosis became more evident. Next, we determined whether cell death following Survivin depletion could be eliminated by preventing mitotic entry. Thymidine (2 mM) was administered along with RNAi, which led to an increased proportion of cells with DNA content between 2 and 4 N and the disappearance of the 4 N peak after 48 h treatment (Fig. 3d), indicating S phase arrest. Notably, after 48 h compound treatment, the level of apoptosis caused by Survivin siRNA was similar to that caused by control siRNA, indicating that administration of thymidine almost completely inhibited the apoptosis caused by Survivin depetion (Fig. 3e). These data suggest that the induction of apoptosis following Survivin depletion was caused by abnormal mitotic progression.

**Figure 4:**
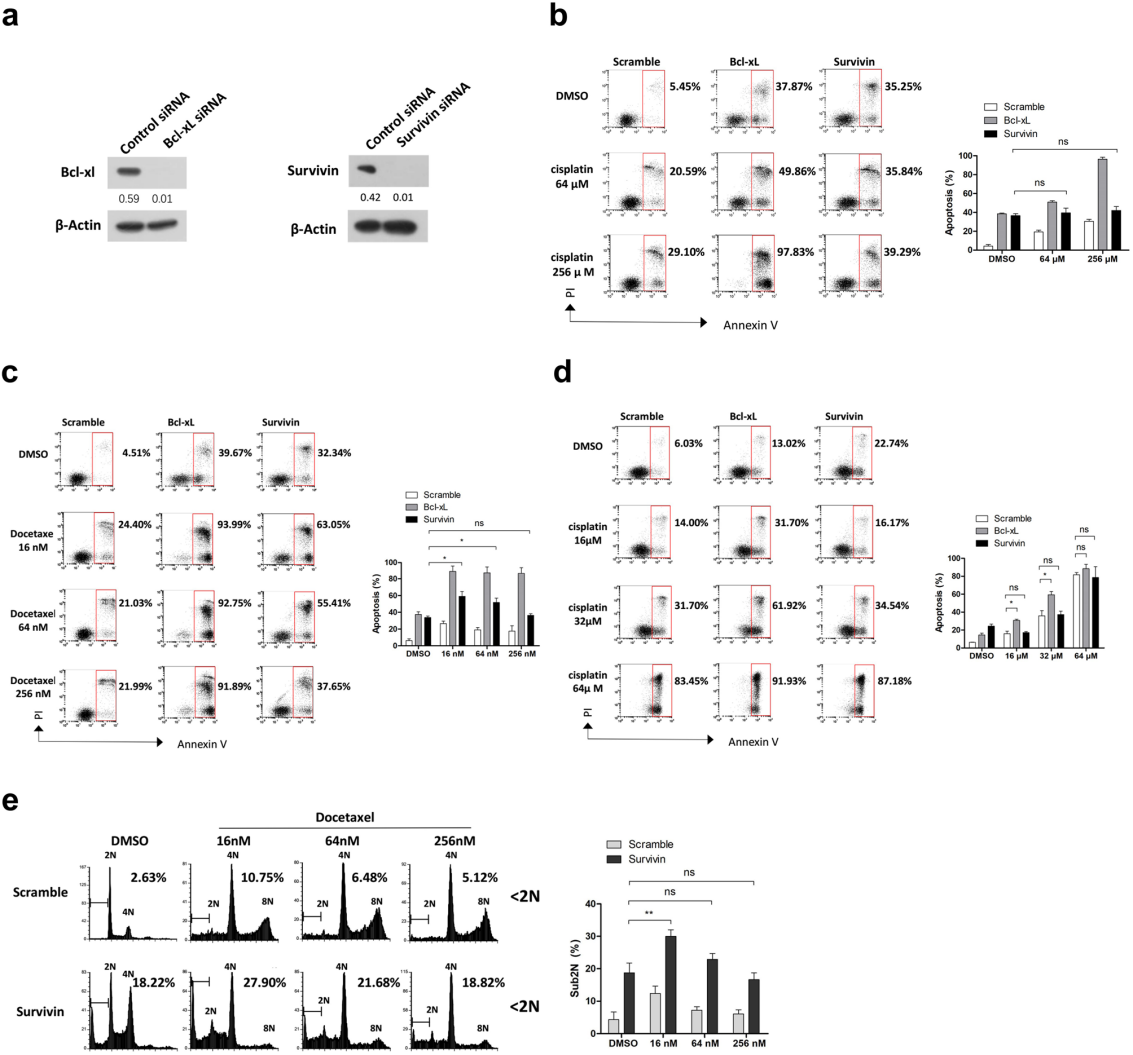
Effects of Survivin depletion on the sensitivity to cisplatin or docetaxel. (**a**) Depletion of Bcl-xl and Survivin by siRNA transfection. MDA-MB-231 cells incubated with the indicated siRNAs for 48 h were harvested and analyzed for protein expression of Bcl-xL or Survivin by western blotting. The band intensities were measured using ImageJ software and normalized to the β-actin. Original blots can be found in Supplementary Fig. S2. (**b-e**) MDA-MB-231 (**b**,**c**,**e**) or HeLa (**d**) cells were transfected with the indicated siRNAs, synchronized with single thymidine arrest, washed and released into fresh medium containing indicated concentrations of cisplatin or docetaxel. Cells were then harvested and analyzed for induction of apoptosis (**b-d**) or cell cycle distribution (**e**). The percentage of apoptotic cells or cells with sub2N DNA content is shown. Values represent mean ± s.d. from a duplicate experiment. *Signifies p < 0.05; **p < 0.01; ns, not significant. Unpaired and two-tailed t test was used.

Survivin function in mitotic regulation is linked with that of the CPC. Inhibition of the Aurora B kinase has also been associated with abnormal mitosis^33^. Consistently, treatment with hesperadin, a small molecule inhibitor of Aurora B^34^, markedly increased the level of multi-nucleation (Fig. 3f) and 4 N population (Fig. 3g). Similar to Survivin depletion, induction of apoptosis was observed in HeLa cells after 36 h treatment with hesperadin (Fig. 3h). To ascertain whether hesperadin-induced apoptosis was associated with aberrant mitosis, thymidine (2 mM) was administered 12 h before hesperadin treatment to prevent entry into mitosis (Fig. 3g). We found the level of apoptosis following hesperidin treatment was reduced (Fig. 3h), indicating that aberrant mitosis contributed to hesperadin-induced cell death. However, administration of thymidine did not completely inhibit hesperadin-induced apoptosis, suggesting that hesperadin may also affect interphase cells. Together, these data demonstrate that apoptosis following Survivin depletion is associated with aberrant mitotic progression, suggesting that the anti-apoptotic function of Survivin is exerted by its role in the CPC.

### Susceptibility of Survivin-depleted cells to docetaxel depends on the drug concentration

Targeting anti-apoptotic Bcl-2 family members or Survivin results in chemosensitization^17, 35^. Here, we compared the level of cell death in Bcl-xL- or Survivin-depleted cells in response to cisplatin or docetaxel treatment. Transfection of siRNA against Bcl-xL or Survivin successfully repressed protein expression in MDA-MB-231 cells (Fig. 4a). Depletion of Survivin results in abnormal mitosis, which may interfere with the action of docetaxel that specifically targets mitotic cells. To minimize this effect, siRNA transfected cells were subjected to single thymidine arrest and then released into DMSO-, cisplatin- or docetaxel-containing media. The concentrations of docetaxel or cisplatin that induced substantial cell death in HeLa cells (> 70%) did not cause massive cell death in MDA-MB-231 cells (< 30%) (Fig. 4b,c), possibly due to the high expression level of Bcl-xL and the tendency of MDA-MB-231 cells to slip out of docetaxel-induced mitotic arrest.

Consistent with previous observations^36^, Bcl-xL depletion substantially increased the cell death induced by cisplatin (Fig. 4b) or docetaxel (Fig. 4c) treatment. Although the level of apoptosis induced by cisplatin was 10–15% higher in Survivin-depleted cells than in control cells, Survivin depletion caused similar level of apoptosis in cisplatin-untreated cells, suggesting that depletion of Survivin does not enhance the sensitivity to cisplatin. Similarly, Bcl-xL depletion enhanced cellular sensitivity of HeLa cells to cisplatin (Fig. 4d). However, cisplatin-induced apoptosis was not significantly increased in Survivin-depleted population. Similar results were obtained in MCF-7 cells (Supplementary Fig. S3).

The effect of Survivin depletion on the sensitivity to docetaxel was more complex. Survivin-depleted MDA-MB-231 cells exhibited elevated level of apoptosis compared with control cells when subjected to 16 or 64 nM docetaxel (Fig. 4c). However, the cell death induced by 256 nM docetaxel was at a similar level to that caused by Survivin depletion alone, showing that the effect of Survivin depletion is dependent on drug concentration. Since docetaxel treatment specifically affects mitotic cells, we compared the cell cycle distribution of Survivn-depleted cells with that of the control cells (Fig. 4e). Docetaxel treatment led to a marked increase of cells with 4 N or 8 N DNA content in the control group, suggesting that cells experiencing an abortive cell division re-duplicate their DNA. In contrast, docetaxel treatment in Survivin-depleted cells resulted in an increase of cells with DNA content that diverged from the 2 N peak, which may reflect loss or gain of DNA content during abnormal cytokinesis^37^. The proportion of cells with sub2N DNA content, reflecting chromosome loss, was proportional to the level of apoptosis (Fig. 4c), suggesting that Survivin depletion may influence the sensitivity to docetaxel by affecting cytokinesis.

### Survivin depletion suppresses docetaxel-induced apoptosis in HeLa cells by facilitating mitotic slippage

**Figure 5:**
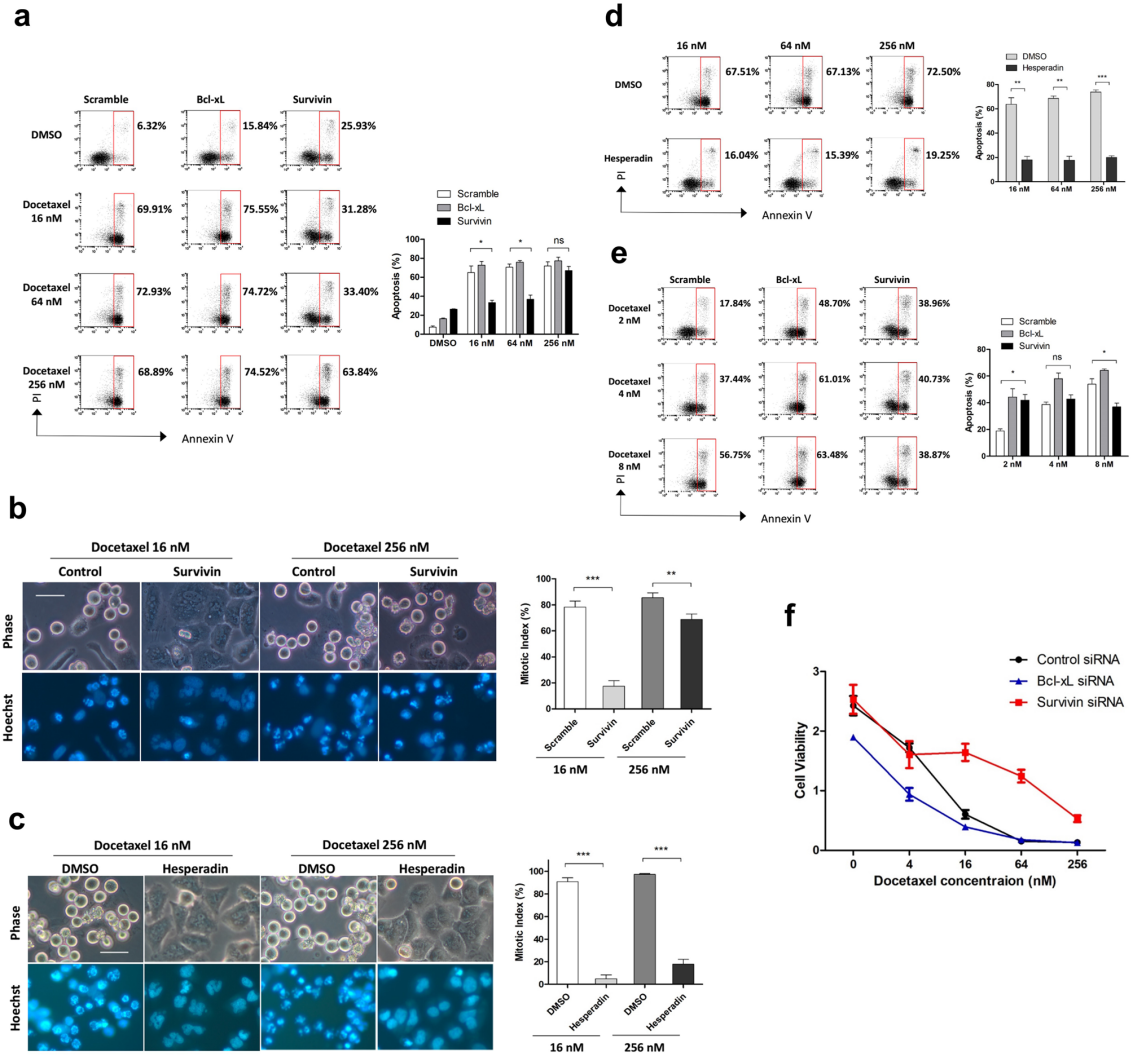
Depletion of Survivin protects HeLa cells against docetaxel treatment. (**a**,**b**) HeLa cells were transfected with the indicated siRNAs, synchronized with single thymidine arrest, washed and released into fresh medium containing either DMSO or indicated concentrations of docetaxel for 24 h and analyzed for induction of apoptosis (**a**). Alternatively, synchronized cells were exposed to the indicated concentrations of docetaxel for 16 h, then stained with Hoechst 33,342 and photographed using phase and Hoechst fluorescence (**b**). (**c**,**d**) HeLa cells were synchronized with single thymidine arrest, washed and released into fresh medium containing the indicated concentrations of docetaxel in combination with either DMSO or Hesperadin (50 nM). Following 16 h combined treatment, cells were stained with Hoechst 33342 and photographed using phase and Hoechst fluorescence (**c**). Alternatively, following 24 h combined treatment, cells were collected and processed for assessment of apoptosis (**d**). (**e**) HeLa cells were transfected with the indicated siRNAs, synchronized with single thymidine arrest, released into medium containing the indicated concentrations of docetaxel for 24 h and analyzed for induction of apoptosis. (**f**) Cell viability assay. HeLa cells were incubated with the indicated siRNAs for 36 h followed by treatment with increasing concentrations of docetaxel for 48 h. Cell viability was determined by using the cck-8 reagent. The percentage of apoptotic or mitotic cells is shown. Values represent mean ± s.d. *Signifies p < 0.05; **p < 0.01; ***p < 0.001; ns, not significant. Unpaired and two-tailed t test was used. Scale bars in (**b**) and (**c**), 50 μm.

To determine whether depletion of Survivin influence the sensitivity to docetaxel in HeLa cells which exhibit robust SAC response, HeLa cells transfected with siRNA against Survivin or Bcl-xL were synchronized with single thymidine arrest and released into fresh media containing DMSO or different concentrations of docetaxel. Depletion of Bcl-xL or Survivin alone yielded ~ 16% or ~ 26% apoptosis, respectively (Fig. 5a). In agreement with previous observations, depletion of Bcl-xL enhanced the cell death induced by docetaxel. However, in contrast to previous reports showing that Survivin inhibition is associated with chemosensitization^17, 38^, depletion of Survivin significantly reduced the fraction of apoptosis caused by 16 or 64 nM docetaxel. The level of apoptosis caused by 256 nM docetaxel was slightly reduced by Survivin depletion.

As Survivin is required for stable SAC response induced by paclitaxel^21^, we investigated whether Survivin-depleted HeLa cells arrested normally following docetaxel treatment. Although most control cells arrested in mitosis following 16 h of treatment with 16 nM docetaxel, Survivin-depleted cells rapidly slipped out of mitosis and became multinucleated (Fig. 5b). However, most of these cells experienced normal mitotic arrest in response to 256 nM docetaxel. As mitotic slippage is frequently linked to taxane resistance^3^, these results may explain why Survivin depletion was more protective against 16 nM docetaxel compared with 256 nM. Inhibition of Aurora B exerted a similar but more potent effect compared with Survivin depletion. Hesperadin treatment efficiently abrogated the mitotic arrest induced by 16 or 256 nM docetaxel (Fig. 5c), and substantially reduced the level of apoptosis caused by either concentration (Fig. 5d), suggesting that Survivin depletion influence cellular response to docetaxel by affecting the function of CPC.

Since 8 nM docetaxel treatment was insufficient to induce extended mitotic arrest, we investigated whether Survivin depletion affected cellular sensitivity to docetaxel at concentrations ≤ 8 nM. Although depletion of Bcl-xL markedly increased the level of apoptosis induced by 2, 4 or 8 nM docetaxel (Fig. 5e), the response of Survivin-depleted cells was more complex. Survivin depletion reduced the cell death caused by 8 nM docetaxel but increased the level of apoptosis caused by 2 nM. However, this does not demonstrate that Survivin depletion enhanced the sensitivity to 2 nM docetaxel, considering that depletion of Survivin alone led to more than 20% apoptosis in HeLa cells. Our results reveal that Survivin depletion was less protective when the concentration of docetaxel was insufficient to induce an extended mitotic arrest, suggesting that the protective effect of Survivin depletion was indeed due to miotic slippage.

To confirm the effect of Survivin depletion on sensitivity to docetaxel, we determined the viability of Survivin-depleted HeLa cells in response to different concentrations of docetaxel by the Cell Counting Kit-8 assay (Fig. 5f). Elevated levels of cell survival were observed in Survivin-depleted cells in response to > 4 nM docetaxel, in contrast to depletion of Bcl-xL, which resulted in drug sensitization. Consistently, Survivin depletion was less protective against 256 nM docetaxel. Survivin depletion also promoted the survival of MCF-7 cells in response to docetaxel treatment (Supplementary Fig. S3).

### Survivin depletion increases cellular senescence and blocks colony formation in the presence of docetaxel

**Figure 6:**
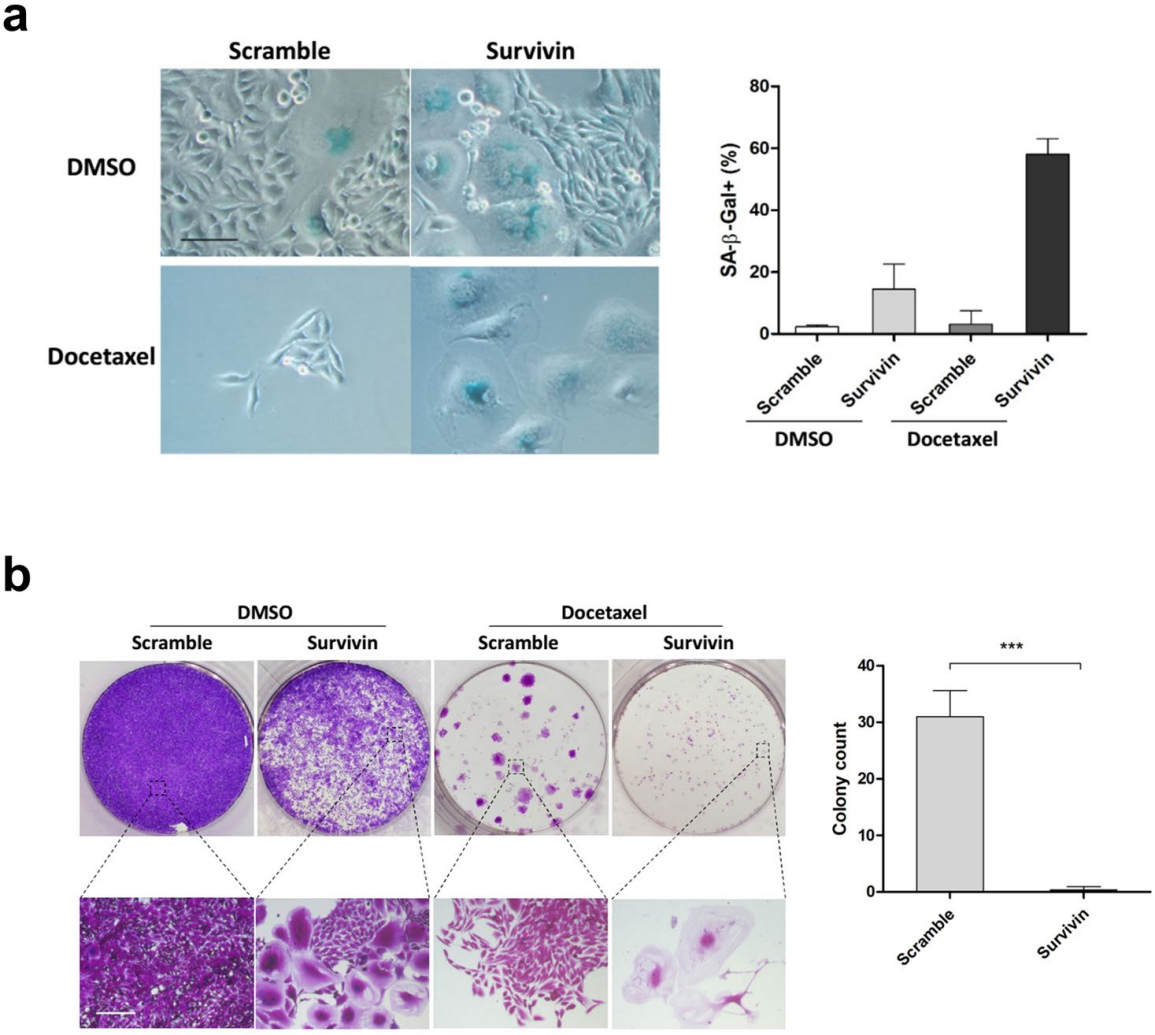
Depletion of Survivin increases docetaxel-induced senescence and suppresses colony formation. (**a**,**b**) HeLa cells were incubated with the indicated siRNAs for 36 h followed by docetaxel treatment for another 36 h, and then incubated in drug-free medium. After 7 days culture without treatment, cells were fixed and the level of cellular senescence was determined by SA-β-Gal staining (**a**). The percentage of SA-β-Gal positive cells is shown. Values represent mean ± s.d. (n = 3 fields). Alternatively, docetaxel-treated cells were cultured in drug-free medium for 2 weeks. Surviving colonies were photographed following fixation with methanol and staining with crystal violet (**b**). Magnified images were obtained under a 10 × objective of microscope. The colony count is shown. Values represent mean ± s.d. from a triplicate experiment. Scale bar in (**a**), 100 μm. Scale bar in (**b**), 200 μm.

Multiple outcomes have been described after cells undergo mitotic slippage, including continued cycling, apoptosis and senescence^39^. Since most Survivin-depleted HeLa cells underwent mitotic death when subjected to 256 nM docetaxel, we followed the fate of these cells after they had slipped out of the mitotic arrest induced by 16 nM Docetaxel. A proportion of cells was found to undergo delayed death, as manifested by the gradually decreased cell number over time. We also observed the accumulation of cells that adopted flattened and enlarged morphology characteristic of cellular senescence, which was confirmed by senescence-associated β-galactosidase (SA-β-Gal) staining (Fig. 6a). Cellular senescence is an irreversible program of cell-cycle arrest that has been identified as a tumor suppressor mechanism alternative to apoptosis^40^. Induction of cellular senescence was also observed in Survivin-depleted cells without docetaxel treatment. Although depletion of Survivin alone failed to inhibit tumor cell growth, as indicated by the rapid proliferation around senescent cells, these cells ceased proliferating when subjected to docetaxel.

To determine the long-term effect of Survivin depletion on cellular response to docetaxel treatment, we performed clonogenic assays of Survivin-depleted HeLa cells. Cells transfected with the Survivin or control siRNA were treated with 16 nM docetaxel for 36 h, and then incubated in drug-free medium. After 2 weeks, surviving colonies were fixed and stained with crystal violet. The results revealed that Survivin-depleted cells did not form colonies when subjected to docetaxel treatment, whereas colony formation of control cells was not completely blocked by docetaxel treatment (Fig. 6b, upper panels). The small spots in the Survivin-depleted population were consistent with sporadic senescent cells, as manifested by magnified images obtained under a 10 × objective of the microscope (Fig. 6b, lower panels), which were in sharp contrast to densely proliferating cells in control group. Together, these results indicate that depletion of Survivin enhances the long-term anti-proliferative effect of docetaxel, possibly by promoting cellular senescence.

## Discussion

Survivin is a bi-functional protein that acts as both an apoptosis inhibitor and a mitotic regulator^9^. Survivin targeting has been associated with tumor cell sensitization to multiple chemotherapeutic agents^17^. However, the sensitivity to taxanes depends on extended mitotic arrest^41^, which requires the normal function of Survivin^21^. Thus, depletion of Survivin can lead to contradictory effects on sensitivity to taxanes. Our data showed that Survivin depletion promoted survival of HeLa cells against docetaxel treatment by facilitating mitotic slippage. Moreover, overexpression of Survivin, or its phosphor-mimetic or deficient mutants via lentiviral transduction did not provide cytoprotection, indicating that its anti-apoptotic function is negligible.

The role of Survivin in apoptosis inhibition has been a subject of controversy^9^. Numerous studies have linked Survivin overexpression to chemoresistance. However, we noticed that most of these observations were based on plasmid transfection^15, 16^ or adenoviral infection^18^, which exhibit dose-dependent toxicity to commonly used cancer cell lines^42, 43^. To circumvent non-specific cytotoxicity, we took advantage of a lentiviral expression system with minimal cytotoxic effects. While adenovirus infection will finally lead to lysis of the target cells, lentiviral vectors generally cause little or no disruption of the target cell even at high titer^44, 45^. In agreement with previous studies, cells transduced with lentiviral particles expressing Bcl-xL or Bcl-2 are evidently more resistant to cisplatin or docetaxel treatment. In contrast, lentiviral overexpression of Survivin or its phospho-mimetic or deficient mutants is not protective. These observations are inconsistent with the anti-apoptotic role of Survivin.

Depletion of Survivin has been linked with spontaneous apoptosis of cancer cells^17^. Consistently, transfection of siRNA against Survivin led to a moderate level of apoptosis (18–35%) in our experimental setting. However, induction of apoptosis was only observed when mRNA levels of Survivin were reduced by more than 90%, which resulted in prominent mitotic abnormalities. The function of Survivin in mitotic regulation is carried out in the context of the CPC. It has been suggested that low levels of Survivin could be able to support significant amounts of kinase activity of Aurora B^46^. To test whether the induction of apoptosis following Survivn depletion results from abnormal mitosis, we blocked the mitotic entry by administration of thymidine, which almost completely eliminated the apoptosis caused by Survivin depletion. Similarly, inhibition of Aurora B led to multi-nucleation and apoptosis, the level of which could be was reduced by prevention of mitotic entry. These results suggest that depletion of Survivin causes cell death through aberrant mitosis by affecting the function of the CPC.

Survivin inhibition has also been associated with sensitization to chemotherapeutic agents^17^. Our results reveal that depletion of Survivin does not seem to sensitize tumor cells to cisplatin, which damages the DNA, but influences the sensitivity to docetaxel, which specifically targets mitotic cells. Survivin depletion increased the level of apoptosis induced by 16 nM docetaxel in the SAC-impaired MDA-MB-231 cells. Cell cycle distribution assay revealed an increased proportion of cells with DNA content that diverged from the 2 N peak, possibly reflecting loss or gain of DNA content during aberrant cytokinesis. However, when subjected to 256 nM docetaxel, Survivin-depleted cells exhibited less sub2N fraction and less apoptosis compared with 16 nM. These results suggest that depletion of Survivin may enhance docetaxel-induced apoptosis by promoting chromosome loss during aberrant cytokinesis.

Contrary to previous reports^17^, we find that depletion of Survivin could provide a protective effect against docetaxel treatment in SAC-proficient HeLa cells, which efficiently arrested and died in mitosis following docetaxel treatment. Although Survivin-depleted HeLa cells arrested normally in the presence of 256 nM docetaxel, they rapidly slipped out of the mitotic arrest cause by 16 nM docetaxel and were significantly more viable than control cells. Similar effects were observed after inhibition of Aurora B. Both Survivin and Aurora B are important for the normal function of the SAC, which is required for sensitivity to taxanes^41^. However, the role of the SAC in the sensitivity to taxanes remains controversial^47^. Inhibition of the SAC kinase Mps1 has been reported to enhance the docetaxel-induced cell death by elevating the frequency of chromosome mis-segregation^48^. We made similar observations that Survivin depletion increased the docetaxel-induced apoptosis in MDA-MB-231 cells. It remains to be determined whether the cell death was associated with chromosome mis-segregation.

Although Survivin-depleted HeLa cells exhibited diminished level of apoptosis following docetaxel treatment, we observed a compensatory increase in the level of cellular senescence. As senescent cells permanently lose the ability to proliferate, cellular senescence has been identified as an alternative tumor suppressor mechanism to apoptosis. However, senescent cells have also been found to promote proliferation of cancer cells through the senescence-associated secretory phenotype (SASP)^49^. To determine the long-term effect of Survivin depletion on docetaxel treatment, we performed clonogenic assay, and the results revealed that depletion of Survivin promoted the efficacy of docetaxel against clonogenic survival of tumor cells. Since docetaxel-induced apoptosis was suppressed by Survivin depletion, it is reasonable to infer that the elevated level of cellular senescence contributes to the anti-proliferative capacity of Survivin depletion.

Together, our results reveal that Survivin depletion can differently affect cellular sensitivity to docetaxel, depending on the cell type and drug concentration. Depletion of Survivin enhances docetaxel-induced apoptosis in SAC-impaired MDA-MB-231 cells, possibly by affecting cytokinesis. However, Survivin depletion suppresses drug-induced apoptosis in SAC-proficient HeLa cells by facilitating mitotic slippage. Moreover, lentiviral overexpression of Survivin is not protective against docetaxel or cisplatin treatment, suggesting that targeting Survivin influences the cell response to docetaxel by affecting mitotic progression rather than sensitizing cells to apoptosis.

## Methods

### Plasmid construction

The coding sequence of Survivin and Bcl-xL were cloned from cDNA of HeLa cells by polymerase chain reaction. The coding sequence of Bcl-2 was base-optimized to reduce the high GC content without changing amino acids and synthesized by Sangon Biotech Co., Ltd (Shanghai, China). The respective coding sequences were first cloned into pcDNA3.1 (Invitrogen, Carlsbad, CA), whereupon site-directed mutagenesis was performed to generate phospho-mimetic or deficient mutants of each gene. The WT or mutant sequences were then cloned into the lentiviral vector pLVX-Puro (Clontech, Mountain View, CA) for lentivirus production. The luciferase gene used for the control was cloned from the pGL3-Basic Vector (Promega, Madison, WI). All inserts were confirmed by DNA sequencing. Primer sequences for PCR amplification are listed in Table 1.

**Table 1:** PCR primers used for vector construction.

Gene	Forward primer (5′–3′)	Reverse primer (5′–3′)
Bcl-2^a^ S70A	CCTGTCGCTAGAACAGCTCCACTGCAG	CTGGGGTCTGCAGTGGAGCTGTTCTAG
Bcl-2^a^ S70E	CCTGTCGCTAGAACAGAACCACTGCAG	CTGGGGTCTGCAGTGGTTCTGTTCTAG
Bcl-xL WT	GGCTCGAGGCCACCATGTCTCAGAGCAACCGG	GGGAATTCTCATTTCCGACTGAAGAGTG
Bcl-xL S62A	TGGCACCTGGCAGACGCGCCCGCGGT	TCCATTCACCGCGGGCGCGTCTGCCAG
Bcl-xL S62E	TGGCACCTGGCAGACGAGCCCGCGGT	TCCATTCACCGCGGGCTCGTCTGCCAG
Survivin WT	GGCTCGAGGCCACCATGGGTGCCCCGACGTTG	GGGAATTCTCAATCCATGGCAGCCAGCT
Survivin T34A	GAGGGCTGCGCCTGCGCGCCGGAGCGG	GGCCATCCGCTCCGGCGCGCAGGCGCA
Survivin T34E	GAGGGCTGCGCCTGCGAGCCGGAGCGG	GGCCATCCGCTCCGGCTCGCAGGCGCA
Luciferase	GGCTCGAGGCCACCATGCATCATCACCATCACCATATGGAAGACGCCAAAAACATAAAG	GGGGATCCTTACACGGCGATCTTTCCGCC

^a^Coding sequence of Bcl-2 has been base optimized to reduce the high GC content.

### Lentivirus production

Lentiviral particles were produced according to the “pLKO.1 Protocol” provided by Addgene (Cambridge, MA). Briefly, WT and mutant sequences of Survivin, Bcl-2, and Bcl-xL were individually cloned into the pLVX-Puro vector. The resultant lentiviral vectors were co-transfected with the packaging plasmid psPAX2 (a gift from Didier Trono, Addgene plasmid # 12260) and envelope plasmid pMD2.G (a gift from Didier Trono, Addgene plasmid # 12259) into 293 T cells using Lipofectamine 2000 (Invitrogen). After 12 h incubation, the medium was replaced with fresh medium. Lentiviral particle-containing medium was harvested from cells after 48 h incubation and filtered through a 0.45-μm filter to remove the 293 T cells, then was directly used to infect target cells.

### Cell culture and drug treatment

HeLa, MDA-MB-231, MCF-7, and 293 T cells were obtained from the Cell Bank of the Chinese Academy of Sciences (Shanghai, China). HeLa and 293 T cells were maintained in Dulbecco’s modified Eagle’s medium (DMEM) (Macgene Technology Ltd., Beijing, China) supplemented with 10% fetal bovine serum (FBS) (Gibco BRL, Life Technologies, Grand Island, NY). MDA-MB-231 cells were grown in L-15 medium (Macgene Technology Ltd.) containing 10% FBS. MCF-7 cells were maintained in DMEM with 10% FBS and 10 μg/ml insulin.

Cell lines stably expressing WT and mutant forms of Survivin, Bcl-2, and Bcl-xL were generated by infection with the indicated lentiviral particles, generated as described above. After selection in medium containing 2 μg/ml puromycin (Macgene Technology Ltd.) for 1 week when the uninfected cells were no longer viable, the infected cells were harvested and overexpression of Survivin, Bcl-2, and Bcl-xL was confirmed by western blot. The expression of mutant protein was confirmed by cDNA pyrosequencing.

### Chemicals

Following chemicals were used for cell treatment, either used alone or combined with other chemicals: docetaxel (Sanofi-Aventis, Gentilli, France), purvalanol A (Tocris, Bristol, UK), cisplatin (Sigma, St. Louis, MO), thymidine (Sigma), hesperadin (MCE, Shanghai, China). 

### Pyrosequencing

Total RNA was isolated from stably transduced cell lines using RNAiso Plus (TaKaRa, Dalian, China) and reverse-transcribed with a PrimeScript™ cDNA Synthesis Kit (TaKaRa) according to the manufacturer’s instructions. Pyrosequencing of each cDNA was performed by Sangon Biotech Co., Ltd.

### Cell synchronization and mitotic index assessment

Synchronization of HeLa or MDA-MB-231 cells was done by a double-thymidine arrest. Cells were treated with 2 mM thymidine for 20 h followed by incubation in thymidine-free medium for 12 h and a second treatment with 2 mM thymidine for 20 h. Alternatively, cells were synchronized by a single 2 mM thymidine arrest for 24 h. After washout cells were subjected to the indicated drug treatment.

At the indicated time points after docetaxel treatment, the cells were detached from the cell culture plate using 0.25% trypsin, resuspended in culture medium containing 10% FBS, cytospinned onto culture plates, fixed with methanol, and stained with Giemsa solution. The percentage of cells exhibiting condensed chromatin was designated as the mitotic index.

### Small interfering RNA transfection

All siRNAs were synthesized by GenePharma Co., Ltd. (Shanghai, China). siRNA sequences from previous reports were used to target Survivin (#1^21^, #2^31^ and #3^32^) or Bcl-xL^50^. siRNA transfection was performed using the Lipofectamine RNAIMAX reagent (Invitrogen) according to the manufacturer’s instructions. The final siRNA concentration was 20 nM.

### Cell viability assay

A Cell Counting Kit-8 (CCK-8) (Dojindo, Kumamoto, Japan) was used to assess cell viability according to the manufacturer’s instructions. Cell viability was determined by measuring the absorbance at 450 nm using a Multiskan FC microplate reader (Thermo Fisher Scientific, Waltham, MA).

### FACS apoptosis assay

Annexin V-FITC/PI staining was performed on fresh cells according to the manufacturer’s specifications (Dojindo). The percentages of apoptotic cells were quantified by combining both early (Annexin V + /PI −) and late (Annexin V + /PI +) apoptotic cells. The flow procedures were performed by using an EPICS XL flow cytometer (Beckman Coulter, Birmingham, UK).

### DNA content analysis

Cells following treatment were collected and fixed with 70% ethanol, and were subsequently incubated with 50 μg/ml propidium iodide (PI) with RNase A at 37 °C for 30 min, and analyzed by FACS. The flow procedures were performed by using an EPICS XL flow cytometer. Cell cycle analysis was carried out by Multicycle software (Phoenix Flow Systems, San Diego, USA).

### Western blot analysis

Cell pellets were lysed in RIPA buffer (Macgene Technology Ltd.) with 10 μl/ml protease inhibitor cocktail (P8340, Sigma) and 10 μl/ml phosphatase inhibitor cocktail (p0044, Sigma). The proteins in cell lysates were separated by electrophoresis on a 15% SDS–polyacrylamide gel, transferred to nitrocellulose membranes, immunostained, and visualized by enhanced chemiluminescence detection reagents (Applygen Technologies Inc., Beijing, China). Antibodies against Survivin, Bcl-2, Bcl-xL, P-Histone 3, phospho-Survivin, and phospho-Bcl-2 were purchased from Cell Signaling (Danvers, MA), and the antibody specific for phospho-Ser-62-Bcl-xL was purchased from Abcam (Cambridge, UK). The β-Actin antibody was purchased from Zsbio (Beijing, China).

### Real-time RT-PCR

Total RNA was isolated using RNAiso Plus (TaKaRa), and reverse-transcribed using PrimeScriptTM RT reagent kit (TaKaRa) according to the manufacturer’s instruction. Real-time PCR was performed using SYBR premix EX Taq (TaKaRa) and analyzed with a CFX96 Touch System (Bio-Rad Laboratories, Hercules, CA). Primers specific for GAPDH and Survivin were designed and synthesized by TaKaRa. The data were represented as mean ± SD from two independent experiments.

### Nuclear staining of mitotic cells

Hoechst 33342 (#4082 Cell Signaling) solution was added to the growth medium of docetaxel-treated cells to obtain a final concentration of 1 μg/ml and incubated at 37 °C for 20 min. Cells were then observed and photographed under a Zeiss Axio Vert.A1 inverted microscope (Zeiss, Germany).

### SA-β-Gal assay

Cells were seeded in 12-well plates. Following treatments, cells were allowed to grow in drug-free medium for one week. Senescent cells were detected by using Senescence β-Galactosidase Staining Kit (#9860 Cell Signaling) following the manufacturer’s instructions.

### Colony formation assays

Cells were seeded in 12-well plates. Following treatments, cells were allowed to grow in drug-free medium for 2 weeks. Surviving colonies were fixed with methanol, and stained by crystal violet. The colony is defined to consist of at least 50 cells.

## Supplementary Information

Supplementary Information.

## Data Availability

The datasets used or analysed during the current study are available from the corresponding author on request.

## References

[1] Montero A, Fossella F, Hortobagyi G, Valero V (2005). Docetaxel for treatment of solid tumours: A systematic review of clinical data. Lancet Oncol..

[2] Gascoigne KE, Taylor SS (2009). How do anti-mitotic drugs kill cancer cells?. J. Cell Sci..

[3] Cheng B, Crasta K (2017). Consequences of mitotic slippage for antimicrotubule drug therapy. Endocr. Relat. Cancer.

[4] Komlodi-Pasztor E, Sackett DL, Fojo AT (2012). Inhibitors targeting mitosis: Tales of how great drugs against a promising target were brought down by a flawed rationale. Clin. Cancer Res..

[5] Gascoigne KE, Taylor SS (2008). Cancer cells display profound intra- and interline variation following prolonged exposure to antimitotic drugs. Cancer Cell.

[6] Manchado E, Guillamot M, Malumbres M (2012). Killing cells by targeting mitosis. Cell Death Differ..

[7] Huang HC, Shi J, Orth JD, Mitchison TJ (2009). Evidence that mitotic exit is a better cancer therapeutic target than spindle assembly. Cancer Cell.

[8] Oltersdorf T (2005). An inhibitor of Bcl-2 family proteins induces regression of solid tumours. Nature.

[9] Mita AC, Mita MM, Nawrocki ST, Giles FJ (2008). Survivin: Key regulator of mitosis and apoptosis and novel target for cancer therapeutics. Clin. Cancer Res..

[10] Altieri DC (2003). Validating survivin as a cancer therapeutic target. Nat. Rev. Cancer.

[11] Carmena, M., Wheelock, M., Funabiki, H. & Earnshaw, W. C. The chromosomal passenger complex (CPC): from easy rider to the godfather of mitosis. Nature reviews. *Mol. cell biol.* **13**, 789–803 (2012). 10.1038/nrm3474 PMC3729939 23175282

[12] Yamamoto H, Ngan CY, Monden M (2008). Cancer cells survive with survivin. Cancer Sci..

[13] O'Connor DS, Wall NR, Porter ACG, Altieri DC (2002). A p34cdc2 survival checkpoint in cancer. Cancer Cell.

[14] Banks DP (2000). Survivin does not inhibit caspase-3 activity. Blood.

[15] Li F (1998). Control of apoptosis and mitotic spindle checkpoint by survivin. Nature.

[16] Tamm, I. *et al.* IAP-family protein survivin inhibits caspase activity and apoptosis induced by Fas (CD95), Bax, caspases, and anticancer drugs. *Cancer Res.* **58**, 5315–5320 (1998). 9850056

[17] Zaffaroni N, Pennati M, Daidone MG (2005). Survivin as a target for new anticancer interventions. J. Cell Mol. Med..

[18] Mesri M, Wall NR, Li J, Kim RW, Altieri DC (2001). Cancer gene therapy using a survivin mutant adenovirus. J. Clin. Invest..

[19] Morikawa, Y. *et al.* Rapamycin enhances docetaxel-induced cytotoxicity in a androgen-independent prostate cancer xenograft model by survivin downregulation. *Biochem. Biophys. Res. Commun.* **419**, 584–589 (2012). 10.1016/j.bbrc.2012.02.089 22387542

[20] Marzia, P. *et al.* Ribozyme-mediated attenuation of survivin expression sensitizes human melanoma cells to cisplatin-induced apoptosis. *J. Clin. Invest.* **109**, 285 (2002). 10.1172/JCI14891 PMC150847 11805141

[21] Ana C, Mar C, Clara S, Earnshaw WC, Wheatley SP (2003). Survivin is required for stable checkpoint activation in taxol-treated HeLa cells. J. Cell Sci..

[22] Masuda A, Maeno K, Nakagawa T, Saito H, Takahashi T (2003). Association between mitotic spindle checkpoint impairment and susceptibility to the induction of apoptosis by anti-microtubule agents in human lung cancers. Am. J. Pathol..

[23] Tao W (2005). Induction of apoptosis by an inhibitor of the mitotic kinesin KSP requires both activation of the spindle assembly checkpoint and mitotic slippage. Cancer Cell.

[24] Terrano DT, Upreti M, Chambers TC (2010). Cyclin-dependent kinase 1-mediated Bcl-xL/Bcl-2 phosphorylation acts as a functional link coupling mitotic arrest and apoptosis. Mol. Cell. Biol..

[25] Ruvolo, P. P., Deng, X. & May, W. S. Phosphorylation of Bcl2 and regulation of apoptosis. *Leukemia* **15**, 515–522 (2001). 10.1038/sj.leu.2402090 11368354

[26] O'Connor DS (2000). Regulation of apoptosis at cell division by p34cdc2 phosphorylation of survivin. Proc. Natl. Acad. Sci. U.S.A..

[27] Arena G (2013). PINK1 protects against cell death induced by mitochondrial depolarization, by phosphorylating Bcl-xL and impairing its pro-apoptotic cleavage. Cell Death Differ..

[28] Barrett, R. M. A., Osborne, T. P. & Wheatley, S. P. Phosphorylation of survivin at threonine 34 inhibits its mitotic function and enhances its cytoprotective activity. *Cell Cycle* **8**, 278–283 (2009). 10.4161/cc.8.2.7587 19158485

[29] Simonian PL, Grillot DA, Nu-Ez G (1997). Bcl-2 and Bcl-XL can differentially block chemotherapy-induced cell death. Blood.

[30] Haschka MD (2015). The NOXA–MCL1–BIM axis defines lifespan on extended mitotic arrest. Nat. Commun..

[31] Chawla-Sarkar M (2004). Downregulation of Bcl-2, FLIP or IAPs (XIAP and survivin) by siRNAs sensitizes resistant melanoma cells to Apo2L/TRAIL-induced apoptosis. Cell Death Differ..

[32] Rödel F (2005). Survivin as a radioresistance factor, and prognostic and therapeutic target for radiotherapy in rectal cancer. Can. Res..

[33] Lens SM, Voest EE, Medema RH (2010). Shared and separate functions of polo-like kinases and aurora kinases in cancer. Nat. Rev. Cancer.

[34] Hauf S (2003). The small molecule Hesperadin reveals a role for Aurora B in correcting kinetochore-microtubule attachment and in maintaining the spindle assembly checkpoint. J. Cell Biol..

[35] Vogler, M., Dinsdale, D., Dyer, M. J. & Cohen, G. M. Bcl-2 inhibitors: small molecules with a big impact on cancer therapy. *Cell Death Differ.* **16**, 360–367 (2009). 10.1038/cdd.2008.137 18806758

[36] Ozvaran, M. K. et al. Antisense oligonucleotides directed at the bcl-xl gene product augment chemotherapy response in mesothelioma. *Mol. Cancer Ther.* **3**, 545–550 (2004). 15141012

[37] Kops GJPL, Foltz DR, Cleveland DW (2004). Lethality to human cancer cells through massive chromosome loss by inhibition of the mitotic checkpoint. Proc. Natl. Acad. Sci..

[38] Olie RA (2000). A novel antisense oligonucleotide targeting survivin expression induces apoptosis and sensitizes lung cancer cells to chemotherapy. Can. Res..

[39] Weaver BA, Cleveland DW (2005). Decoding the links between mitosis, cancer, and chemotherapy: The mitotic checkpoint, adaptation, and cell death. Cancer Cell.

[40] Collado M, Serrano M (2010). Senescence in tumours: Evidence from mice and humans. Nat. Rev. Cancer.

[41] Sudo T, Nitta MH, Ueno NT (2004). Dependence of paclitaxel sensitivity on functional spindle assembly checkpoint. Can. Res..

[42] Lv H, Zhang S, Wang B, Cui S, Yan J (2006). Toxicity of cationic lipids and cationic polymers in gene delivery. J. Control. Release.

[43] Brand K, Klocke R, Possling A, Paul D, Strauss M (1999). Induction of apoptosis and G2/M arrest by infection with replication-deficient adenovirus at high multiplicity of infection. Gene Ther..

[44] Carlotti F (2004). Lentiviral vectors efficiently transduce quiescent mature 3T3-L1 adipocytes. Mol. Ther. J. Am. Soc. Gene Ther..

[45] Hong S (2007). Functional analysis of various promoters in lentiviral vectors at different stages of in vitro differentiation of mouse embryonic stem cells. Mol. Ther. J. Am. Soc. Gene Ther..

[46] Yue Z (2008). Deconstructing Survivin: Comprehensive genetic analysis of Survivin function by conditional knockout in a vertebrate cell line. J. Cell Biol..

[47] Yamada HY, Gorbsky GJ (2006). Spindle checkpoint function and cellular sensitivity to antimitotic drugs. Mol. Cancer Ther..

[48] Maia AR (2015). Inhibition of the spindle assembly checkpoint kinase TTK enhances the efficacy of docetaxel in a triple-negative breast cancer model. Ann. Oncol..

[49] Faget, D. V, Ren, Q. & Stewart, S. A. Unmasking senescence: context-dependent effects of SASP in cancer. *Nat. Rev. Cancer* **19**, 439–453 (2019). 10.1038/s41568-019-0156-2 31235879

[50] Bruey, J. M. et al. Bcl-2 and Bcl-XL regulate proinflammatory caspase-1 activation by interaction with NALP1. *Cell* **129**, 45–56 (2007). 10.1016/j.cell.2007.01.045 17418785

