# Supplementary material for: Depletion of Survivin suppresses docetaxel-induced apoptosis in HeLa cells by facilitating mitotic slippage
Source: Sci Rep. 2021 Jan 27;11:2283. doi: 10.1038/s41598-021-81563-3 (PMC7840972; doi:10.1038/s41598-021-81563-3)
Supplement: Supplementary file 1 — Supplementary Information. [file 41598_2021_81563_MOESM1_ESM.docx]

Supplementary Information for

**Depletion of Survivin suppresses docetaxel-induced apoptosis in HeLa cells by facilitating mitotic slippage**

Teng-Long Han^1^, Hang Sha^1^, Jun Ji^1^, Yun-Tian Li^1^, Deng-Shan Wu^1^, Hu Lin^1^, Bin Hu^1^ and Zhi-Xin Jiang^1^

^1^The 305 Hospital of the People's Liberation Army, Beijing 100017, China. Correspondence and requests for materials should be addressed to T.L.H.(hantenglong@hotmail.com) or Z.X.J.([658jzx@163.com](mailto:658jzx@163.com)).

**This PDF file includes:**

Figs. S1 to S3

**Supplementary Figure S1: Western blots cropped for Figure 2a (2), 2b (4) and 2e (3)**

**(2a1) Western blot: Bcl-2 and Bcl-xL**


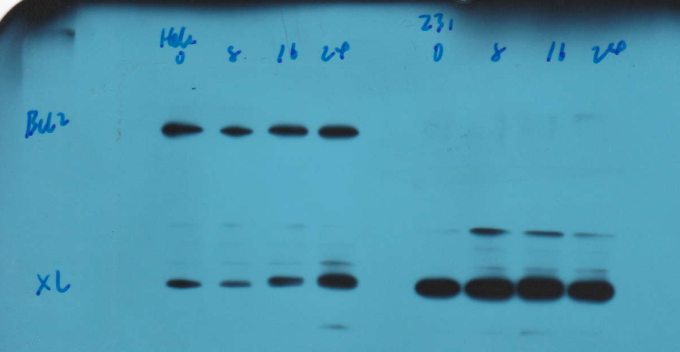


**(2a2) Western blot: β-Actin and Survivin**


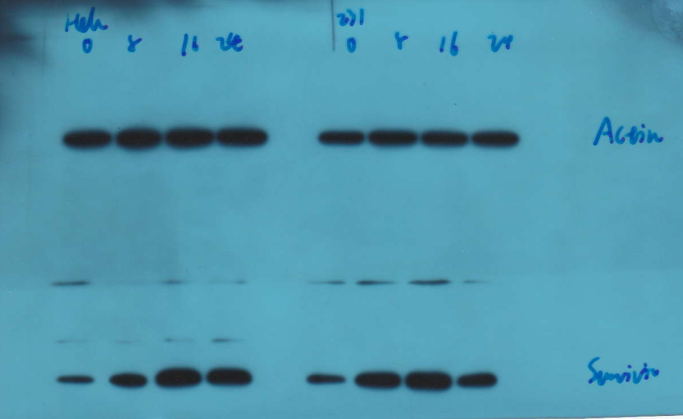


**(2b1) Western blot: p-Histone H3 and p-Survivin**


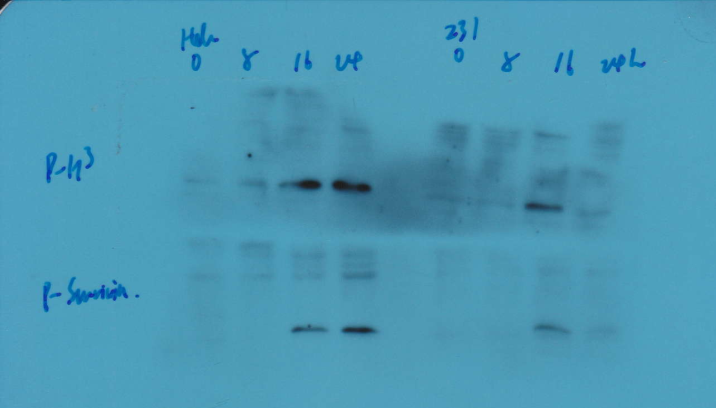


**(2b2) Western blot: p-Bcl-2**


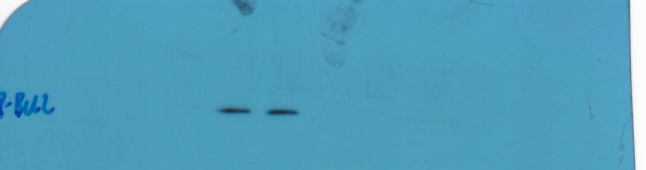


**(2b3) Western blot: p-Bcl-xL**


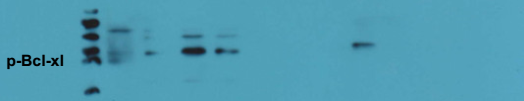


**(2b4) Western blot: β-Actin**


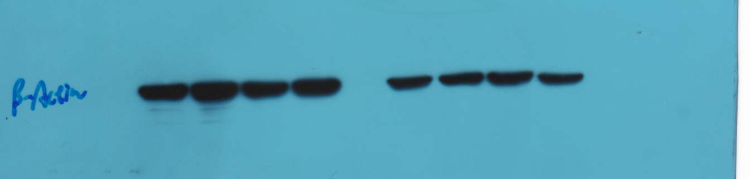


**(2e1) Western blot: Bcl-2 and β-Actin**


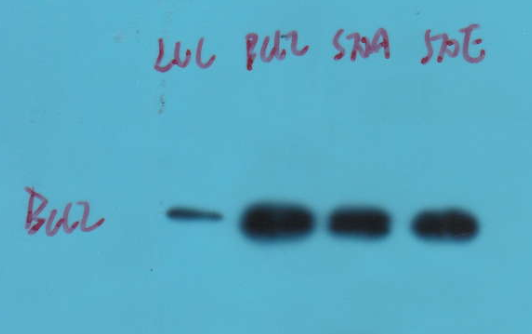

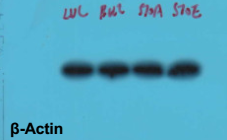


**(2e2) Western blot: Bcl-xL and β-Actin**


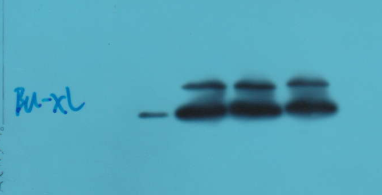

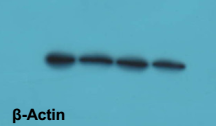


**(2e3) Western blot: β-Actin and Survivin**


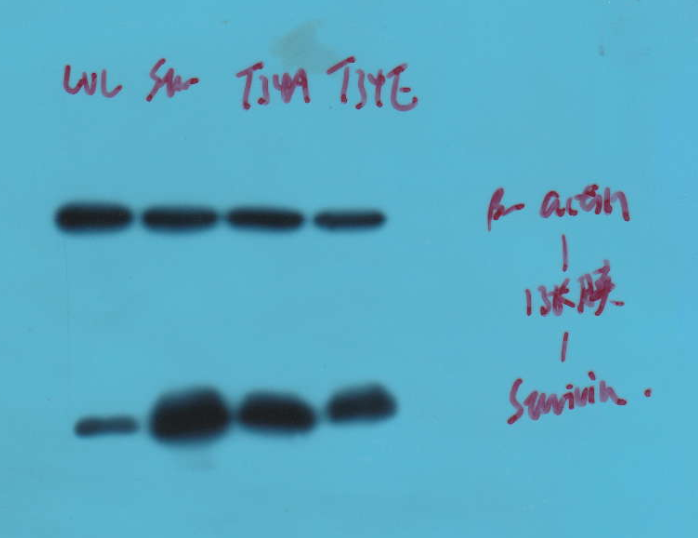


**Supplementary Figure S2: Western blots cropped for Figure 4a (2)**

**(4a1) Western blot: β-Actin and Bcl-xL**


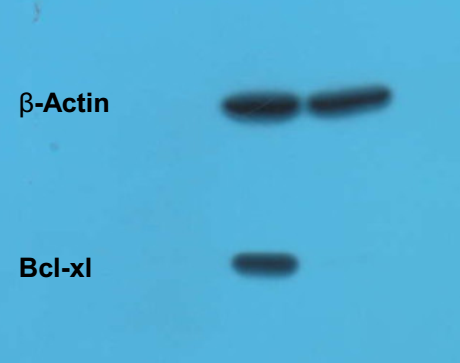


**(4a2) Western blot: β-Actin and Survivin**


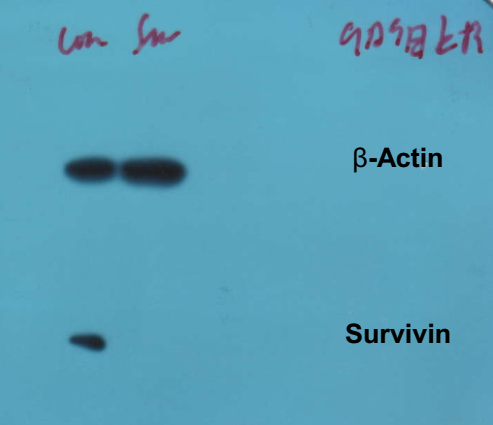


**Supplementary Figure S3: Depletion of Survivin does not increase the sensitivity to cisplatin but promotes cell survival against Docetaxel.**


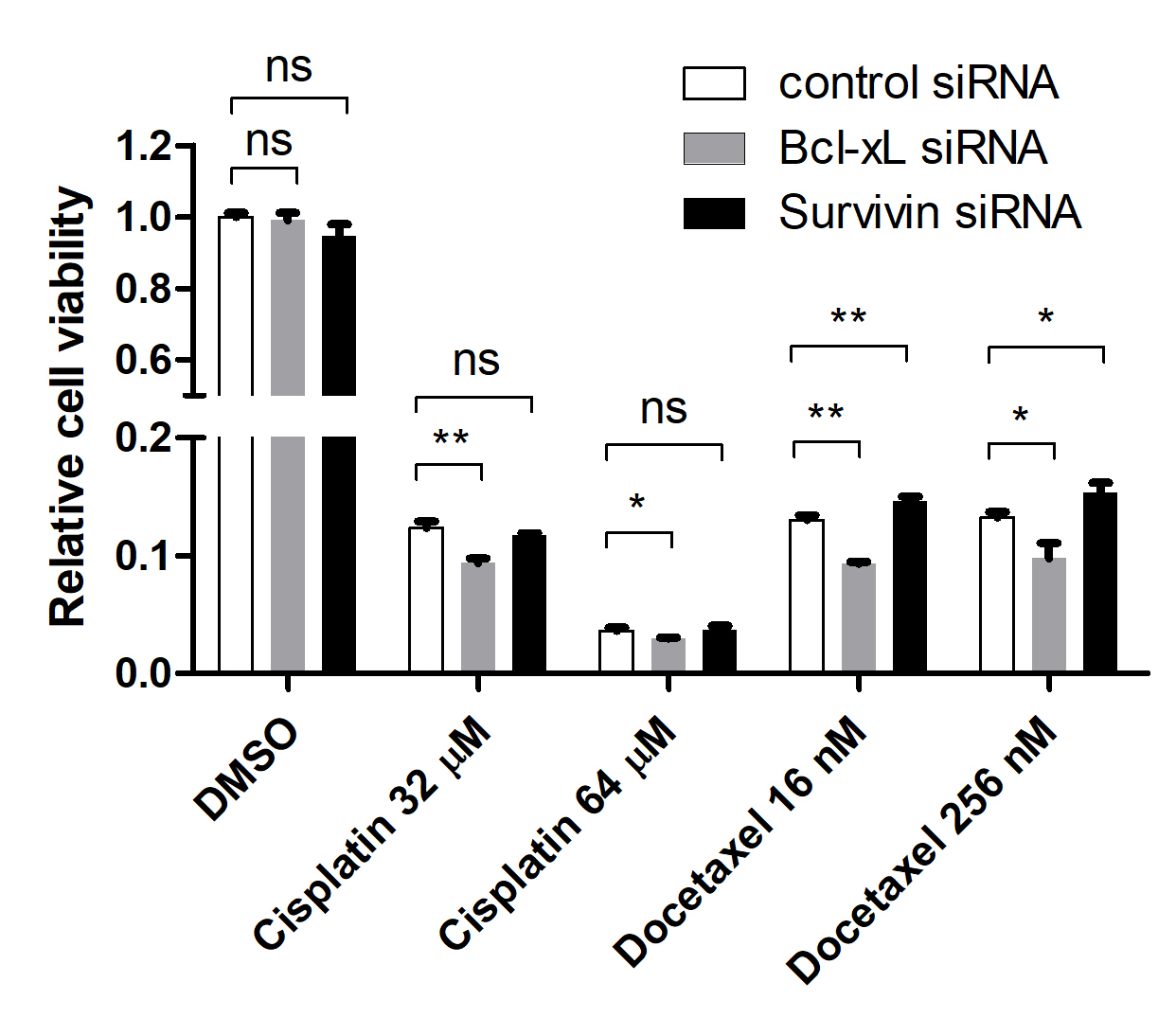


MCF-7 cells were incubated with the indicated siRNAs for 24 h followed by docetaxel or cisplatin treatment for 6 days. Cell viability was determined by using the cck-8 reagent. Values represent mean ± s.d. (n = 3 wells). * signifies p< 0.05; ** p< 0.01; ns, not significant. Unpaired and two-tailed t test was used.
